# Dual Disruption of EGFR/PI3K Signaling: IGF2BP2 Targeting Reverses Anti-EGFR Resistance in CAFs-Infiltrated Oral Squamous Cell Carcinoma

**DOI:** 10.3390/ijms26093941

**Published:** 2025-04-22

**Authors:** Yaying Hu, Tianshuang Zhu, Sheng Nong, Yanan Sun, Yiwei Li, Junchen Pan, Jiyuan Ma, Jiali Zhang

**Affiliations:** 1 State Key Laboratory of Oral & Maxillofacial Reconstruction and Regeneration, Key Laboratory of Oral Biomedicine Ministry of Education, Hubei Key Laboratory of Stomatology, School & Hospital of Stomatology, Wuhan University, Wuhan 430079, China; yaying_hu@whu.edu.cn (Y.H.); 2020283040063@whu.edu.cn (T.Z.); 2019303041010@whu.edu.cn (S.N.); yanansun@whu.edu.cn (Y.S.); liyiwei8690@163.com (Y.L.); 2021203040003@whu.edu.cn (J.P.); majiyuan@whu.edu.cn (J.M.); 2 Department of Oral Pathology, School and Hospital of Stomatology, Wuhan University, Wuhan 430079, China

**Keywords:** IGF2BP2, EGFR/PI3K/AKT, PIK3R1, m6A, RBPs, *Igf2bp2*-deficient mice, cancer-associated fibroblasts, cetuximab

## Abstract

RNA-binding proteins (RBPs) critically regulate post-transcriptional gene networks, yet their roles and mechanisms in oral squamous cell carcinoma (OSCC) remain underexplored. Dysregulated RBPs were identified through integrated analysis of RNA-seq and single-cell RNA-seq. The oncogenic functions of IGF2BP2 were evaluated through tissue microarrays, CCK-8, transwell assays, mouse xenografts, and *Igf2bp2*-deficient mouse models of tongue SCC (TSCC). Subsequently, we utilized RNA-seq, RIP-seq, RIP/MeRIP-qPCR, and dual-luciferase reporter assays to investigate IGF2BP2-target genes. Furthermore, cell co-culture system and mouse TSCC models were used to validate the therapeutic effect of the IGF2BP2 inhibitor. IGF2BP2 was the most markedly upregulated RBP in OSCC cells and cancer-associated fibroblasts (CAFs), correlating with unfavorable prognosis. IGF2BP2 deprivation significantly impaired human OSCC proliferation and metastasis, and delayed mouse TSCC onset. Mechanistically, IGF2BP2 stabilized EGFR and PIK3R1 mRNA via m6A-dependent interactions, thereby sustaining activation of the EGFR/PI3K/AKT oncogenic axis. Pharmacological inhibition of IGF2BP2 exhibited anti-OSCC efficacy in vivo and in vitro by concurrently suppressing EGFR and PI3K/AKT pathway activity, overcoming anti-EGFR resistance resulting from cell-intrinsic PI3K/AKT hyperactivation and CAF-secreted factors. Our findings identified IGF2BP2 as a master regulator of OSCC progression and a promising therapeutic target, offering an alternative strategy for OSCC patients suffering anti-EGFR resistance.

## 1. Introduction

RNA-binding proteins (RBPs) constitute a class of proteins that dynamically interact with and regulate RNA metabolism, exerting a pivotal role in gene regulation at the post-transcriptional level [1,2]. Accumulating evidence indicates that RBPs critically regulate multiple oncogenic processes, including tumor cell metabolism, immune escape, proliferation, invasion, and migration [3,4]. However, the molecular characterization of RBPs in oral squamous cell carcinoma (OSCC) progression remains inadequately elucidated. In our investigation, we identified insulin-like growth factor 2 mRNA binding protein 2 (IGF2BP2) as the most markedly upregulated RBP in tumor cells and cancer-associated fibroblasts (CAFs).

IGF2BP2 is a member of the insulin-like growth factor 2 mRNA binding protein family, involved in RNA localization, stabilization, and translation [5]. It plays a critical role in various cancer progressions [6,7,8], functioning as an m6A modification reader that modulates mRNA stability and translation efficiency [9,10]. Although current studies provide evidence that elevated IGF2BP2 levels in OSCC tissues are associated with poor prognosis [11], the specific mechanisms by which IGF2BP2 contributes to OSCC development remain largely unknown. To the best of our knowledge, previous studies have identified several target genes in OSCC, including c-Myc, SLC7A11, HK2, slug, and LB1CC1 [11,12,13,14,15]. In this research, we elucidate two novel IGF2BP2 target genes, epidermal growth factor receptor (EGFR) and phosphoinositide-3-kinase regulatory subunit 1 (PIK3R1).

EGFR, a well-established therapeutic target in tumor progression [16], is amplified in 80–90% of OSCC cases and linked to poor outcomes [17]. The upregulation of EGFR leads to autophosphorylation of EGFR, subsequently activating multiple oncogenic signaling cascades, including the Ras/MAPK, PI3K/AKT, and STAT pathways, which collectively accelerate malignant tumor biological functions [18,19]. Cetuximab-based chemotherapy, a monoclonal antibody targeting EGFR, is widely used in clinical practice for OSCC, but its therapeutic efficacy is often limited by the development of both intrinsic and acquired resistance [20]. Activation of bypass signaling (via amplification or mutations of HER2, FGFR1, MET, and PDGFR) or downstream signaling (via mutations of NRAS, BRAF, PIK3CA, or deletion of PTEN) in cancer cells has been shown to contribute to resistance to anti-EGFR antibodies [21]. Beyond cell-autonomous resistance, CAFs, as key components of the tumor microenvironment (TME), have been discovered to mediate resistance to anti-EGFR therapy in a context-dependent manner through increasing EGF and IGF-binding protein secretion [22,23].

Compensatory activation of the PI3K/AKT pathway has been proposed as a major driver of resistance to anti-EGFR treatment in HNSCC [24]. PIK3R1, encoding the major regulatory subunit of PI3K, has been found to enhance resistance to gefitinib in lung cancer, one of the EGFR tyrosine kinase inhibitors [25]. However, the functional role of PIK3R1 in mediating resistance to anti-EGFR antibodies, particularly in the context of OSCC, remains unexplored. Although combination therapies of targeting EGFR and PI3K/AKT pathway inhibitors have shown promise, the associated side effects from two or more drugs limit their clinical applicability [24]. Therefore, the development of single agents capable of simultaneously targeting EGFR and PI3K represents a promising therapeutic strategy with potentially improved safety profiles.

In this study, we present novel insights that IGF2BP2 is essential for EGFR and PIK3R1 mRNA stability and activation of the EGFR/PI3K/AKT axis in OSCC tumor cells and CAFs. Targeting IGF2BP2 effectively overcomes anti-EGFR-resistance mediated by either intracellular PI3K/AKT signaling hyperactivation or CAF-mediated tumor microenvironment interactions. These may offer a promising alternative therapeutic strategy for OSCC patients suffering from anti-EGFR treatment resistance.

## 2. Results

### 2.1. IGF2BP2 Is the Most Markedly Upregulated RBP in OSCC Cells and CAFs, and Is Associated with Poor Clinical Prognosis

**Figure 1:**
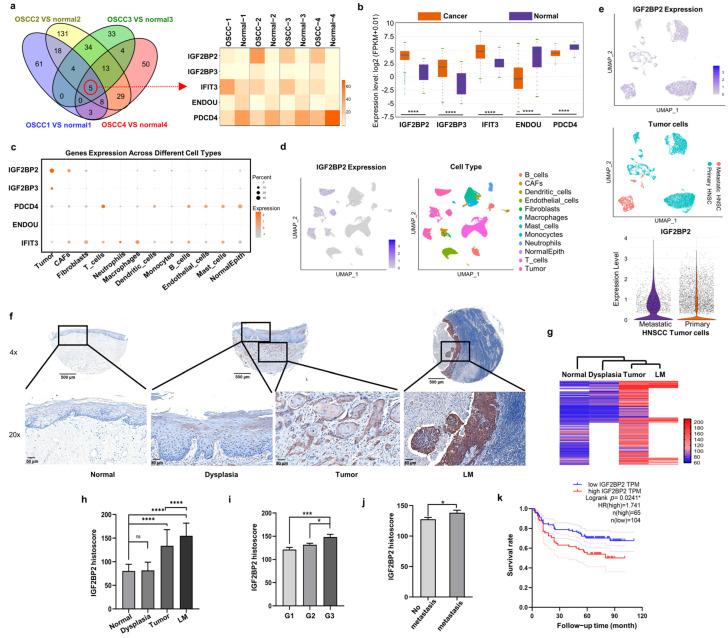
IGF2BP2 is the most markedly upregulated RNA-binding protein (RBP) in OSCC cells and CAFs, and is associated with poor clinical prognosis. (**a**) Dysregulated RBPs in OSCC tissues identified by RNA-seq. Heat map illustrated the expression of co-dysregulated RBPs. (**b**) Expression of five RBPs in HNSCC based on the TCGA database. (**c**,**d**) Expression of five RBPs in different cell types based on scRNA-seq. (**e**) Uniform manifold approximation and projection (UMAP) visualization of primary and metastatic HNSCC highlighted different IGF2BP2 expression. (**f**) Representative images of IGF2BP2 staining in various OSCC tissues. LM: lymph node metastasis. (**g**) Results of hierarchical clustering analysis visualized IGF2BP2 expression patterns across different tissues. (**h**) Histoscores of IGF2BP2 in different tissues. *n* = 196. (**i**,**j**) IGF2BP2 expression was associated with tumor grade (*n* = 195) and lymph node metastasis (*n* = 196). G1/G2/G3: grade 1/grade 2/grade 3. (**k**) Kaplan–Meier survival analysis of OSCC patients. * *p* < 0.05, *** *p* < 0.001, **** *p* < 0.0001, ^ns^ *p* > 0.05.

To unveil dysregulated RBPs in OSCC, we conducted RNA-seq on paired cancerous and adjacent normal tissues from four patients. Notably, IGF2BP2, IGF2BP3, and IFIT3 were consistently upregulated, while ENDOU and PDCD4 were uniformly downregulated across all cancerous tissues (Figure 1a). To validate these findings, we analyzed the expression of these genes in HNSCC tissues using TCGA RNA-seq data via StarBase v3.0 (Figure 1b). The scRNA-seq dataset from 22 HNSCC tissues demonstrated that IGF2BP2 had the highest expression abundance in tumor cells and CAFs compared with other four RBPs or other cell types (Figure 1c,d). Results of further analysis revealed a dramatic increase in IGF2BP2 expression in metastatic HNSCC tumor cells compared with primary HNSCC tumor cells (Figure 1e).

We assessed the correlation between IGF2BP2 levels and clinicopathological characteristics of OSCC patients using tissue microarrays (Figure 1f), with detailed statistical analyses presented in Table S1. Results of the hierarchical cluster analysis clearly distinguished OSCC and lymph node metastasis (LM) groups from other tissue groups (Figure 1g). Results of the statistical analysis demonstrated that IGF2BP2 protein levels in OSCC cells were significantly higher than in dysplastic and normal epithelial cells, yet lower than those in metastatic lymph nodes (Figure 1h). Moreover, higher IGF2BP2 expression was observed in poorly differentiated tumor (Figure 1i) and lymph node metastasis (Figure 1j). Results of the Kaplan–Meier survival analysis of our collected samples indicated that patients with higher IGF2BP2 levels were significantly associated with unfavorable 5-year survival (Figure 1k).

### 2.2. Impaired IGF2BP2 Expression Inhibits Tumor Progression in OSCC

**Figure 2:**
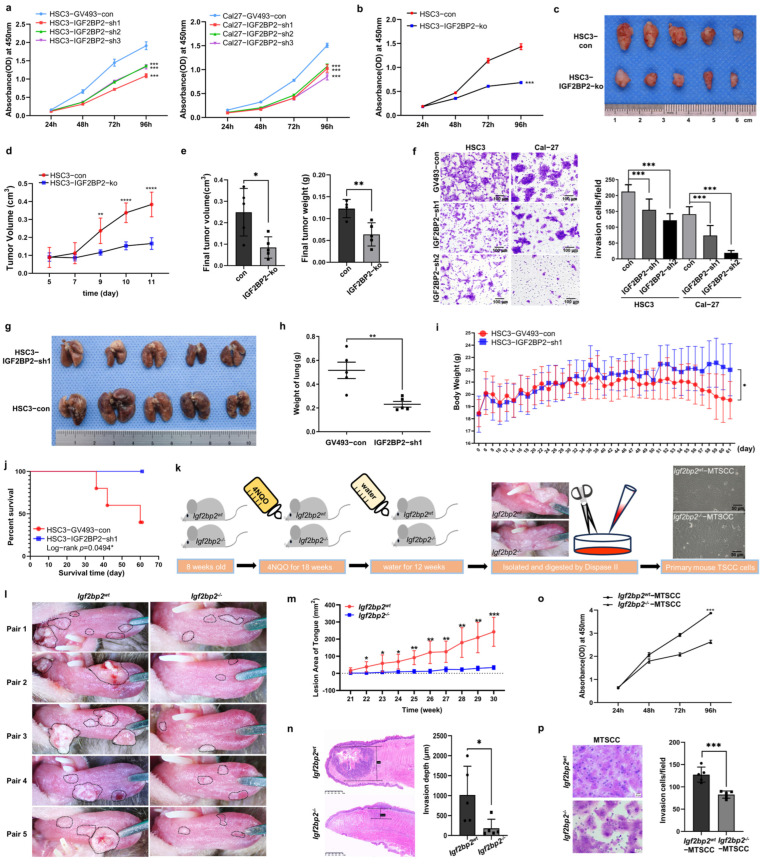
Impaired IGF2BP2 expression inhibits tumor progression in OSCC. (**a**) Inhibition of IGF2BP2 reduced cell growth in HSC3 and Cal-27 cells. *n* = 3. (**b**) Stable IGF2BP2 knockout in HSC3 cells markedly inhibited cell growth. *n* = 3. (**c**) Images of tumors from nude mice bearing OSCC xenografts. (**d**,**e**) Growth curves, volumes, and weights of xenograft tumors. *n* = 5. (**f**) Cell invasion was suppressed in HSC3 and Cal-27 cells after IGF2BP2 knockdown. *n* = 3. (**g**–**j**) Images and weights of lung tissues, mice body weights, and survival rates from nude mice injected with HSC3–GV493-con/IGF2BP2-sh1 cells. *n* = 5. (**k**) Schematic diagram of the mouse TSCC model induction and primary mouse TSCC cell culture methodology. (**l**) Images of tongues from 4NQO-treated *Igf2bp2^wt^* and *Igf2bp2^−/−^* mice. *n* = 5. The lesioned area is delineated by a black dashed line. (**m**) Growth curves of the lesion area. (**n**) Infiltration depth of TSCC. (**o**,**p**) Proliferation and invasion of primary mouse TSCC cells. *n* = 3, * *p* < 0.05, ** *p* < 0.01, *** *p* < 0.001, **** *p* < 0.0001.

To investigate the role of IGF2BP2 in tumorigenesis, we established OSCC cell lines with IGF2BP2 knockdown or knockout and confirmed their efficiency by Western blot (Figure S1). Downregulated IGF2BP2 significantly reduced proliferation in HSC3 and Cal-27 cells (Figure 2a). On the other hand, based on the sensitivity to cetuximab (data from the website of Genomics of Drug Sensitivity in Cancer), HSC3 (sensitive) and Cal-27 (resistant) cell lines were selected for further research. IGF2BP2 knockout in HSC3 cells resulted in a greater than 50% reduction in cell growth (Figure 2b). In subcutaneous xenograft models, tumors in the HSC3–IGF2BP2-ko group exhibited significantly slower growth kinetics compared with those in the control group (Figure 2c–e). Transwell assays revealed a substantial diminishment in the invasive potential of IGF2BP2-deficient HSC3 and Cal-27 cells (Figure 2f). Furthermore, compared with the control cohort, mice injected with IGF2BP2-knockdown HSC3 cells exhibited strikingly reduced lung metastasis formation (Figure 2g,h), accompanied by mitigated body weight loss (Figure 2i), and improved survival outcomes (Figure 2j).

To further investigate the crucial role of *Igf2bp2* in carcinogenesis, we established TSCC models in C57BL/6N mice with different *Igf2bp2* genotypes, *wild-type* (*Igf2bp2*^wt^) and *homozygous knockout* (*Igf2bp2*^−/−^), through the administration of 4NQO-containing water (Figure 2k). Following equivalent exposure durations, *Igf2bp2*^wt^ mice developed multiple white patches, ulcers, or cauliflower-like nodules on their tongues, while *Igf2bp2*^−/−^ counterparts exhibited milder symptoms (Figure 2l). Notably, *Igf2bp2*^wt^ mice manifested earlier onset and faster progression of TSCC compared with the *Igf2bp2*^−/−^ mice (Figure 2m). Results of histopathological analysis demonstrated a significant difference in TSCC infiltration depths between the two genotypes (Figure 2n). Moreover, primary TSCC cells derived from *Igf2bp2*^−/−^ mice unveiled a significant reduction in the proliferation and invasion ability compared with those from *Igf2bp2^wt^* mice (Figure 2o,p).

### 2.3. IGF2BP2 Regulates EGFR and PIK3R1 in an m6A-Dependent Manner

**Figure 3:**
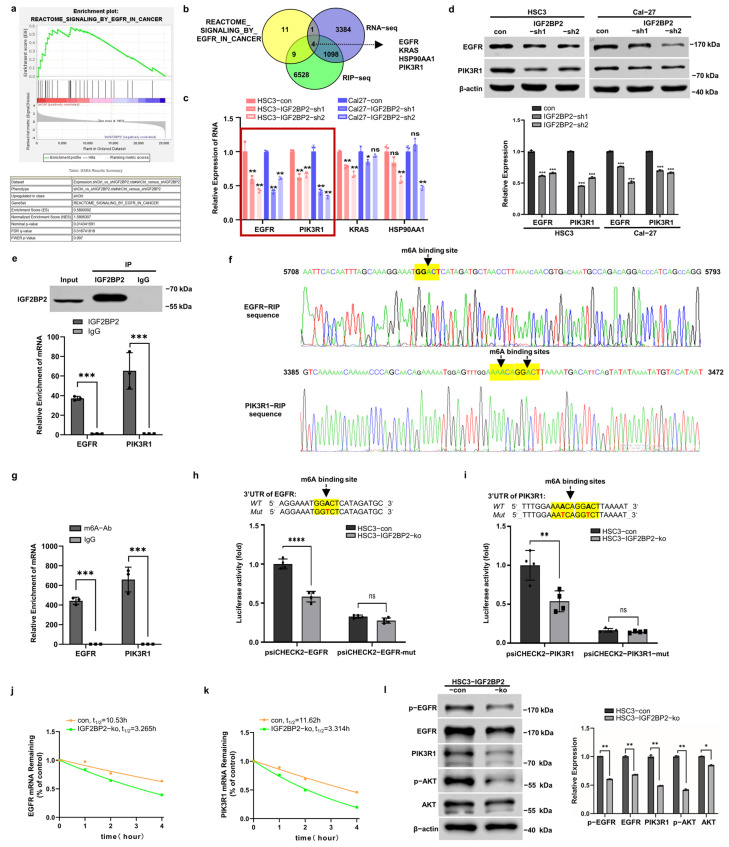
IGF2BP2 regulates EGFR and PIK3R1 in an m6A-dependent manner. (**a**) Results of GSEA analysis of RNA-seq revealed the enrichment of REACTOME_SIGNALING_BY_ EGFR_IN_CANCER pathway-related genes correlating with IGF2BP2 expression. (**b**) Venn diagram illustrating the overlap between pathway-enriched genes, RNA-seq, and anti-IGF2BP2 RIP-seq datasets. (**c**) RNA expression levels of EGFR, PIK3R1, KRAS, and HSP90AA1 in HSC3 and Cal-27 cells after IGF2BP2 knockdown. The data were normalized to the mRNA expression of β-actin. *n* = 3. (**d**) The protein expression of EGFR and PIK3R1 were suppressed by IGF2BP2 downregulation. The data were normalized to the protein expression of β-actin. *n* = 3. (**e**) RIP-qPCR assays confirmed the binding sites between IGF2BP2 and 3′UTR of EGFR and PIK3R1 mRNA. *n* = 3. (**f**) Schematics illustrated the EGFR-RIP sequence and PIK3R1-RIP sequence with very high-confidence m6A sites predicted by SRAMP software (version 2016). (**g**) Results of MeRIP-qPCR analysis confirmed the m6A sites. *n* = 3. (**h**,**i**) Luciferase reporter assays measured the luciferase activities of 3′UTR-wt or 3′UTR-mut of EGFR and PIK3R1 in HSC3 cells with IGF2BP2 knockout. *n* = 4. (**j**,**k**) EGFR and PIK3R1 mRNA decay rates in HSC3-IGF2BP2-ko cells. *n* = 3. (**l**) Protein expression levels of EGFR, p-EGFR, PIK3R1, AKT, and p-AKT in HSC3-con/HSC3-IGF2BP2-ko cells. *n* = 3. * *p* < 0.05, ** *p* < 0.01, *** *p* < 0.001, **** *p* < 0.0001, ^ns^ *p* > 0.05.

To identify potential target RNAs of IGF2BP2, we performed RNA-seq on HSC3-con/IGF2BP2-sh cells, followed by RIP-seq. Results of the gene set enrichment analysis (GSEA) revealed that the enrichment of EGFR_IN_CANCER pathway-related genes correlated with IGF2BP2 expression (Figure 3a), with four candidate targets identified in both RIP-seq and RNA-seq datasets (Figure 3b). Among the candidate targets, EGFR and PIK3R1 mRNA expressions showed the most pronounced reduction following IGF2BP2 knockdown in both HSC3 and Cal-27 cells (Figure 3c). Consistent with the mRNA findings, EGFR and PIK3R1 protein expressions were significantly altered in IGF2BP2-knockdown cells (Figure 3d).

Subsequent confirmation via RIP-qPCR revealed a significant enrichment of specific 3′UTR fragments of EGFR and PIK3R1 mRNA in the anti-IGF2BP2 group compared with the IgG control (Figure 3e). Furthermore, specific IGF2BP2-binding sequences were identified through mRNA-seq, and within which highly confident m6A sites were predicted using SRAMP (Figure 3f). MeRIP-qPCR assays validated the putative m6A sites, showing higher enrichment of EGFR and PIK3R1 mRNA segments in the anti-m6A group (Figure 3g). To functionally characterize the m6A sites, we mutated the core m6A sites and constructed luciferase reporter vectors harboring the wild-type 3′UTR (3′UTR-wt) or mutated 3′UTR (3′UTR-mut) sequences of EGFR and PIK3R1 mRNA. Dual-luciferase reporter gene assays indicated a significant attenuation of luciferase activity induced by 3′UTR-wt in HSC3–IGF2BP2-ko cells compared with controls (Figure 3h,i). In contrast, 3′UTR-mut constructs showed similarly low luciferase activity in both control and IGF2BP2-knockout cells.

RNA stability assays demonstrated significantly shortened half-lives of both EGFR and PIK3R1 mRNA in IGF2BP2-knockout cells (Figure 3j,k). Furthermore, a notable decrease in protein levels of EGFR, phosphorylated EGFR (Y1068), PIK3R1, and phosphorylated AKT (S473) was detected in HSC3–IGF2BP2-knockout cells, indicating the critical role of IGF2BP2 in regulating the EGFR/PI3K/AKT axis (Figure 3l).

### 2.4. IGF2BP2 Inhibitor Exhibits Potent Anti-OSCC Efficacy in Both Cetuximab-Sensitive and -Resistant Cells

**Figure 4:**
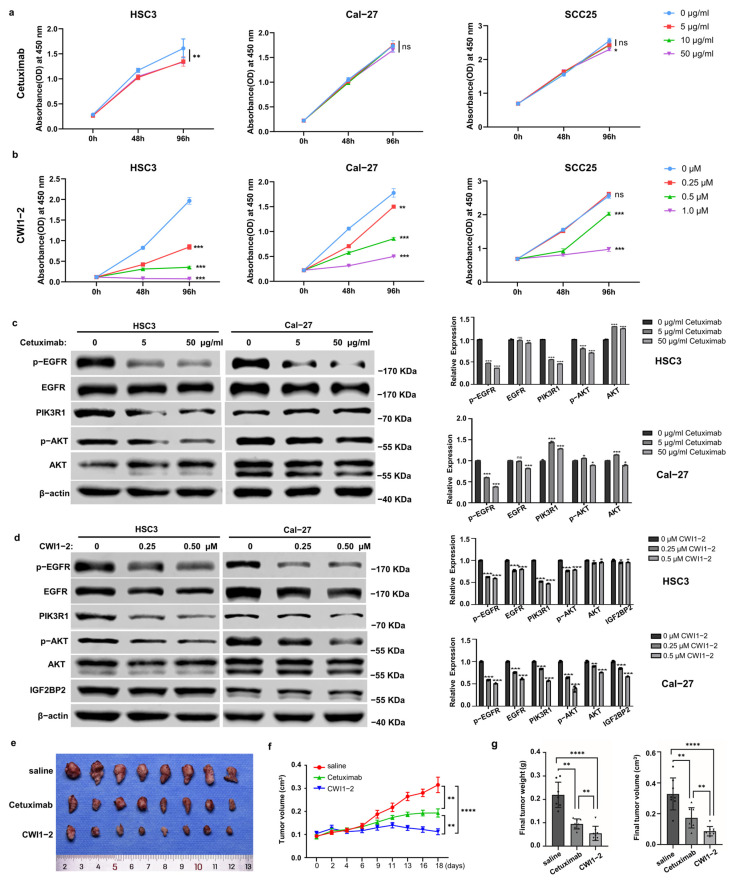
IGF2BP2 inhibitor exhibits potent anti-OSCC efficacy in both cetuximab-sensitive and -resistant cells. (**a**,**b**) Cell growth of HSC3, Cal-27, and SCC25 treated with cetuximab or CWI1-2. *n* = 3. (**c**,**d**) Protein expression levels of EGFR, p-EGFR, PIK3R1, p-AKT, and AKT in HSC3 and Cal-27 cells treated with cetuximab or CWI1-2 for 48 h. *n* = 3. (**e**) Image of xenograft tumors of Cal-27 from mice treated with saline, cetuximab, or CWI1-2. *n* = 8. (**f**,**g**) Growth curves, volumes, and weights of xenograft tumors. * *p* < 0.05, ** *p* < 0.01, *** *p* < 0.001, **** *p* < 0.0001, ^ns^ *p* > 0.05.

To compare the therapeutic efficacy of the IGF2BP2 inhibitor (CWI1-2) and cetuximab, we conducted cell viability assays using CCK-8. Cetuximab significantly reduced the proliferation of HSC3 cells, indicating cetuximab sensitivity, but showed minimal effects on Cal-27 and SCC25 cells, suggesting potential cetuximab resistance (Figure 4a). On the other hand, CWI1-2 significantly inhibited the proliferation of HSC3 (cetuximab-sensitive) and Cal-27 and SCC25 (cetuximab-resistant) cells in a dose-dependent manner (Figure 4b). Subsequently, we found that cetuximab reduced p-EGFR, PIK3R1, and p-AKT levels in HSC3 cells, but PI3KR1 expression remained hyperactivated in Cal-27 cells, indicating compensatory PI3K/AKT pathway activation in cetuximab-resistant cells (Figure 4c). Notably, CWI1-2 effectively suppressed EGFR/PI3K/AKT signaling in both cell lines, irrespective of their cetuximab sensitivity status (Figure 4d). Next, we evaluated the in vivo antitumor efficacy of CWI1-2 and cetuximab using Cal-27 xenograft models. CWI1-2 treatment significantly reduced tumor proliferation rates, volumes, and weights compared with both control and cetuximab-treated cohorts (Figure 4e–g).

### 2.5. CWI1-2 but Not Cetuximab Suppresses EGFR/PI3K/AKT Pathway in CAFs

**Figure 5:**
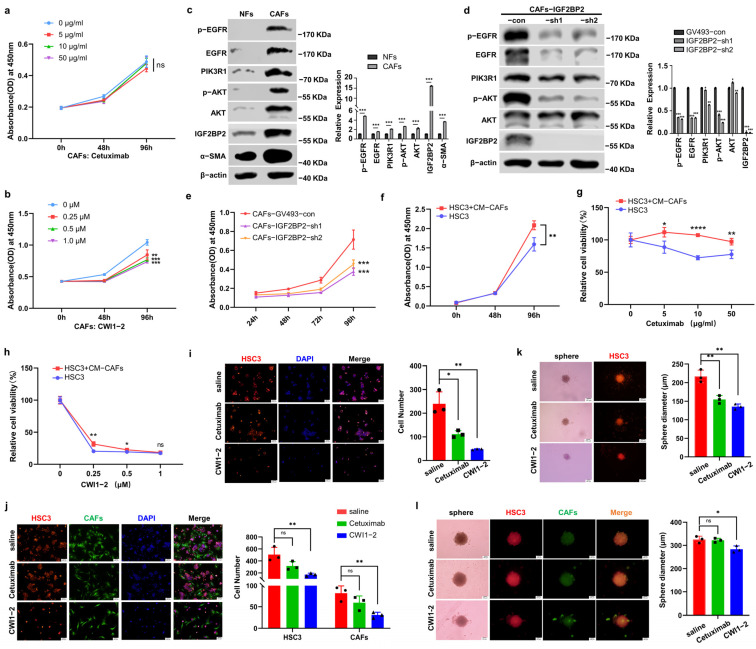
CWI1-2 overcomes CAFs-mediated cetuximab resistance in OSCC. (**a**,**b**) Growth of CAFs treated with cetuximab or CWI1-2. *n* = 3. (**c**,**d**) Protein expression of α-SMA, EGFR, p-EGFR, PIK3R1, p-AKT, AKT, and IGF2BP2 in NFs and CAFs (**c**), and in CAFs with IGF2BP2 knockdown (**d**). *n* = 3. (**e**) Cell growth in CAF-con/CAF-IGF2BP2-shs cells. *n* = 3. (**f**). Conditioned medium from CAFs (CM-CAFs) enhanced the proliferation of HSC3 cells. *n* = 3. (**g**,**h**) Therapeutic efficacy of cetuximab (**g**) or CWI1-2 (**h**) in HSC3 cells in the presence of CM-CAFs. *n* = 3. (**i**,**j**) Representative immunofluorescence images of HSC3 cells monoculture (**i**) or co-cultured with CAFs (**j**) in the presence of saline, cetuximab, or CWI1-2. Bar charts depicted cell numbers in each group. *n* = 3. (**k**,**l**) Representative images of tumor spheres from each group are given. Bar charts displayed the sphere diameter across groups. *n* = 3. * *p* < 0.05, ** *p* < 0.01, *** *p* < 0.001, **** *p* < 0.0001, ^ns^ *p* > 0.05.

CAFs are well-established mediators of tumor progression and resistance to anti-EGFR therapies [26,27]. Firstly, we successfully isolated and characterized primary normal fibroblasts (NFs) and CAFs from adjacent normal tissues and OSCC, confirmed by α-SMA and FAP (Figure S2). CCK-8 assays revealed that the proliferation of CAFs remained unaffected by cetuximab but was significantly inhibited by CWI1-2 (Figure 5a,b). Consistent with scRNA-seq analysis, CAFs exhibited significantly higher IGF2BP2 expression and enhanced EGFR/PI3K/AKT pathway activation compared with NFs (Figure 5c). Impaired IGF2BP2 attenuated pathway activation (Figure 5d) and reduced CAFs proliferation (Figure 5e).

### 2.6. CWI1-2 Overcomes CAFs-Mediated Cetuximab Resistance in OSCC

We further investigated the pharmacological effects of CWI1-2 and cetuximab independently on tumor cells within CAF-infiltrated tumor microenvironments. When cultured in a conditioned medium from CAFs (CM-CAFs), HSC3 cells exhibited significantly accelerated proliferation (Figure 5f) and developed notable cetuximab resistance (Figure 5g), while maintaining sensitivity to CWI1-2 treatment (Figure 5h). In direct co-culture systems, the efficacy of cetuximab on HSC3 cell was discouraged, while the efficacy of CWI1-2 was sufficient to effectively inhibit the growth of both HSC3 cells and CAFs (Figure 5i,j). Although both cetuximab and CWI1-2 significantly inhibited spheroid formation in monoculture conditions (Figure 5k), the presence of CAFs in co-culture systems completely abolished cetuximab-mediated inhibition of tumor spheroid proliferation (Figure 5l). Notably, CWI1-2 retained robust suppression of spheroid formation even under CAF-supported tumor conditions, highlighting its microenvironment-resistant therapeutic profile (Figure 5l).

### 2.7. CWI1-2 Exhibits Promising Anti-Tumor Efficacy in 4NQO-Induced TSCC Mouse Model

**Figure 6:**
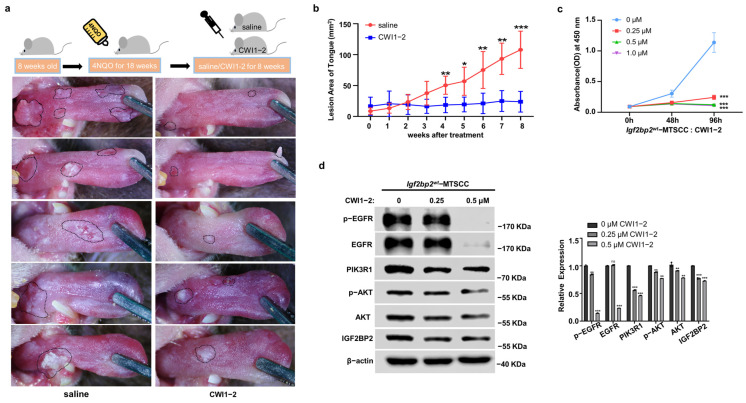
CWI1-2 exhibits promising anti-tumor efficacy in 4NQO-induced TSCC mouse model. (**a**) Images of tongues from mice treated with saline or CWI1-2. *n* = 5. (**b**) Growth curves of lesion area delineated by a black dashed line. (**c**) CWI1-2 inhibited the growth of *Igf2bp2^wt^*-MTSCC cells. *n* = 3. (**d**) Protein expression of EGFR, p-EGFR, PIK3R1, p-AKT, and AKT in *Igf2bp2^wt^*-MTSCC cells treated with CWI1-2. *n* = 3. * *p* < 0.05, ** *p* < 0.01. *** *p* < 0.001.

In the 4NQO-induced TSCC model of *Igf2bp2*^wt^ mice, CWI1-2-treated mice displayed milder symptoms and reduced lesion growth kinetics on their tongues compared with untreated counterparts (Figure 6a,b). In vitro experiments demonstrated that CWI1-2 treatment significantly inhibited the proliferation and EGFR/PI3K/AKT axis activation in *Igf2bp2*^wt^-MTSCC cells (Figure 6c,d).

## 3. Discussion

RBPs have been extensively documented to play pivotal roles in various aspects of tumor progression [28]. A comprehensive study identified 1542 RBPs across 15 cancer types [29], highlighting their widespread involvement in oncogenesis. In our study, we identified five co-dysregulated RBPs, among which IGF2BP2 exhibited the most pronounced expression variability and the highest expression levels in both cancer cells and CAFs. This aligns with a pan-cancer landscape bioinformatics analysis showing elevated IGF2BP2 expression in HNSCC [30], suggesting its potential as a robust therapeutic target.

Recent experimental findings have linked IGF2BP2 to overall survival in OSCC patients [31]. Nevertheless, the expression dynamics of IGF2BP2 across various stages of epithelial carcinogenesis remain inadequately characterized. Here, we demonstrate that elevated IGF2BP2 expression is predominantly confined to cancer cells, rather than dysplastic epithelial cells, suggesting its potential utility as a diagnostic biomarker for OSCC. Our study employing TSCC models in transgenic mice demonstrated that the absence of *Igf2bp2* precipitates several notable ramifications. Firstly, deprivation of *Igf2bp2* leads to a delay in the onset of tongue mucosal cancer, signifying a postponement in the initiation of tumorigenesis. Secondly, it ensues in a deceleration of carcinogenesis, implying a decelerated growth and spread of cancer cells. Lastly, it correlates with a lower degree of malignancy, indicating reduced aggressiveness and potential for metastasis in the developed tumors. Collectively, these findings underscore the critical role of *Igf2bp2* in driving the initiation and progression of OSCC.

As an m6A reader, IGF2BP2 has been reported to maintain mRNA stability by binding to their 3′UTR, CDs, or the junction between the 3′UTR and CDs [9,15,32,33]. It has also been implicated in regulating multiple oncogenic processes in OSCC, including metastasis, autophagy, and metabolic reprogramming, through its regulation of key targets such as c-Myc, SLC7A11, HK2, slug, and LB1CC1 [11,12,13,14,15]. In this study, we demonstrate that IGF2BP2 is involved in EGFR_IN_CANCER signaling as a stabilizer of m6A-modified EGFR and PIK3R1 mRNA by targeting their 3′UTR regions, providing novel insights into the molecular mechanisms underlying IGF2BP2 function in OSCC.

EGFR amplification, occurring in 80–90% of HNSCC cases, drives ligand-independent receptor phosphorylation, a critical event in tumor progression [17,18,19,34,35,36]. EGFR phosphorylation initiates the activation of multiple downstream signaling cascades, including Ras-Raf-MAPK, PI3K/AKT, and JAK/STAT pathways [18,37]. Our findings reveal that EGFR/PI3K/AKT signaling is significantly attenuated in IGF2BP2*^−/−^* human OSCC cells and *Igf2bp2^−/−^* mouse TSCC cells. This signaling axis has been known to not only promote OSCC oncogenesis but also compromise the efficacy of anti-EGFR therapies [38,39].

Cetuximab-based chemotherapy, specifically targeting EGFR, is a mainstay treatment for HNSCC, but few patients benefit from it because of intrinsic resistance or acquired resistance after prolonged treatment [40,41,42,43]. Therefore, elucidating the molecular mechanisms underlying resistance and developing innovative therapeutic strategies to overcome cetuximab resistance are critical unmet needs. Our study demonstrates that cetuximab resistance in OSCC cells is associated with PIK3R1 upregulation and subsequent hyperactivation of the PI3K/AKT pathway. PIK3R1 encodes P85α, a crucial regulatory subunit of PI3K, and plays intricate roles in tumor progression and drug resistance [44]. Our findings reveal a previously unrecognized role of PIK3R1 in mediating cetuximab resistance, expanding its known functions in therapeutic resistance to various agents, including cisplatin, platinum compounds, gemcitabine, anti-PD-1 therapy, and gefitinib [25,45,46,47,48,49].

It is now well accepted that resistance to anti-EGFR therapies arises from both cell-autonomous mechanisms and non-cell-autonomous interactions between cancer cells and TME [26,50,51]. CAFs, one of the most abundant components within TME, promote tumor malignancy and confer anti-EGFR therapy resistance through multiple mechanisms, including ECM remodeling, secretion of soluble molecules, exosome vesicle delivery, and metabolic crosstalk [52]. CAFs have been documented to modulate resistance to cetuximab by secreting excessive epidermal growth factor or matrix metalloproteinases [22,53]. Consistent with these mechanisms, our experimental data demonstrate that both CAFs and CAF-educated OSCC cells exhibit significant resistance to cetuximab. Furthermore, CAFs exhibit elevated IGF2BP2 expression and enhanced EGFR/PI3K/AKT pathway activation compared with normal fibroblasts, and silencing IGF2BP2 effectively suppresses CAFs proliferation and inhibits pathway activation. Our findings suggest that IGF2BP2 may serve as critical molecular mediators of cancer cell–CAF crosstalk, although the precise mechanisms warrant further investigation.

Collectively, these above findings establish IGF2BP2 as a promising therapeutic target for overcoming cetuximab resistance. Given the critical role of PI3K/AKT signaling, the combination of anti-EGFR agents and PI3K inhibitors has been considered as a therapeutic strategy [24]. However, the increased toxicity associated with combination therapies presents significant clinical challenges that need to be addressed. Therefore, we propose CWI1-2, a novel small-molecule IGF2BP2 inhibitor, as a superior therapeutic alternative due to its dual targeting of EGFR and PIK3R1 signaling with reduced toxicity compared with combination therapies. Notably, CWI1-2 demonstrates potent anti-tumor activity against both cetuximab-sensitive and -resistant OSCC cells, as well as CAFs and CAF-infiltrated tumor cells. Furthermore, it was found to significantly attenuate carcinogenesis in 4NQO-induced mouse TSCC models. Hence, CWI1-2, by targeting the endogenous IGF2BP2/EGFR/PI3K/AKT axis, represents a novel therapeutic approach that overcomes EGFR-targeted resistance attributed to both autonomous and non-cell-autonomous interactions between cancer cells and CAFs. Moreover, CWI1-2 has also been shown to effectively inhibit IGF2BP2 protein expression and demonstrates potent anti-leukemia efficacy [54]. These findings highlight the broad therapeutic potential of CWI1-2 across multiple tumor types.

CWI1-2 suppresses IGF2BP2 activity by competitively inhibiting its binding to RNA targets, resulting in broad inhibition of IGF2BP2–m6A interactions across transcripts. Our current study has unequivocally established CWI1-2’s potent suppression of the IGF2BP2/EGFR/PI3K/AKT axis in both OSCC cells and CAFs. However, there are several critical aspects unresolved in this study and warrant further investigation, including the dynamic crosstalk between OSCC cells and CAFs following CWI1-2 treatment and the comprehensive elucidation of pharmacological mechanisms of CWI1-2.

## 4. Materials and Methods

### 4.1. Clinical Samples Collection

OSCC samples for RNA-seq (4 fresh) and tissue microarrays (196 paraffin-embedded) were collected at Stomatology Hospital of Wuhan University with ethics approval. The histological types and grades were verified by two pathologists.

### 4.2. Cell Culture, Plasmid Construction, and Transfection

Cal-27, SCC25, and HEK-293E cells were obtained from ATCC. HSC3 cells were generously provided by Professor Qianming Chen. Cells were cultured in a DMEM (Cal-27, HSC3), DME/F12 (SCC25), or RPMI (HEK-293E) medium with 10% FBS.

IGF2BP2 knockdown was achieved using lentiviral vectors. GV493-IGF2BP2-sh1/2/3 (Genechem, Shanghai, China) sequences were listed in Table S2. Lentiviruses were packaged in HEK-293E cells using lipofectamine 3000 (Invitrogen, Carlsbad, CA, USA). Generation of HSC3-IGF2BP2-ko cells was detailed in Figure S3.

### 4.3. RNA Sequencing (RNA-Seq) and RNA Immunoprecipitation Sequencing (RIP-Seq)

RNA-seq followed protocols in our previous study [55]. RIP-seq was commissioned to ABLife (Wuhan, China). In brief, HSC3 cells were lysed and incubated with protein A/G immunomagnetic beads pre-conjugated with IGF2BP2 antibody. Subsequently, the IGF2BP2-bound RNA was isolated from beads. The cDNA libraries were then prepared and sequenced using the Hi-seq 2000 system.

### 4.4. Western Blotting, RT-qPCR, Immunohistochemistry (IHC), and Immunofluorescence (IF) Staining

Western blotting, RT-qPCR, IHC, and IF were performed as previously described [56]. Primary antibodies used: β-actin, IGF2BP2, EGFR (Proteintech, Wuhan, China), p-EGFR, AKT, p-AKT (Cell Signaling Technology, Danvers, MA, USA), α-SMA, PI3K (Abclonal, Wuhan, China), and pan-CK (ZSGB-Bio, Beijing, China). Primer sequences for RT-qPCR were listed in Table S3.

### 4.5. CCK-8, Transwell Assay, and Mice Xenografts

CCK-8, transwell assay, and xenografts in BALB/c nude mice were conducted as previously described [55,56]. In vivo, 1 × 10^6^ cells were inoculated via tail vein for metastasis, while 1 × 10^7^ cells were subcutaneously inoculated for proliferation. To study therapeutic efficacy, once the subcutaneous xenograft volumes reached 100 mm^3^, the mice were intraperitoneally administered with cetuximab (2.0 mg/kg, MCE, Monmouth Junction, NJ, USA) or CWI1-2 (5.0 mg/kg, MedMol, Shanghai, China) three times per week.

### 4.6. Primary Cell Culture, Purification, and Passage from Human and Mouse TSCC and Adjacent Normal Tissues

Human and mouse TSCC tissues were minced and digested with 1 mg/mL Dispase II (Roche, Basel, Switzerland) and 0.2 mg/mL DNase I (BioFroxx, Einhausen, Germany) for 3 h, and the filtered supernatants were transferred for primary culture. Differential trypsinization method was used to purify fibroblasts and epithelial cells, and repeated at each cell passage [57]. Spindle-shaped fibroblasts, more sensitive to trypsin, detached first, leaving behind epithelial cells. The mouse TSCC cell lines have been subcultured for over 30 passages, consistently retaining stable morphology.

Normal fibroblasts (NFs) were obtained from histologically normal tissues ≥ 1 cm from the tumor margin, and CAFs were isolated from tumor cores. CAFs were identified by significantly elevated expression of α-smooth muscle actin (α-SMA) and fibroblast activation protein (FAP) compared with NFs.

### 4.7. Cell Co-Culture Models

For sphere formation assay, HSC3 cells (1 × 10^3^, pre-labeled with red fluorescent probes, Invitrogen, CA, USA) and CAFs (2 × 10^3^, stably transfected with GFP) were inoculated into ultra-low attachment surface 96-well plates (CORNING, Corning, NY, USA), supplying with SM1, b-EGF, and EGF. Resulting tumor spheres were treated with cetuximab (50 μg/mL) or CWI1-2 (0.2 μM) for 9 days. For cell count co-culture experiments, HSC3 cells (6 × 10^3^) and CAFs (1.2 × 10^4^) were inoculated into 24-well plates and treated with cetuximab (50 μg/mL) or CWI1-2 (0.2 μM) for 3 days. Images were captured using a fluorescent microscope (OLYMPUS IX83, OLYMPUS, Tokyo, Japan).

### 4.8. RNA Stability Assay

Cells were cultured in 12-well plates overnight and treated with 5 μg/mL actinomycin D (MCE, NJ, USA). RNA was extracted at various time points for quantification.

### 4.9. RNA Immunoprecipitation-qPCR (RIP-qPCR) and Methylated RNA Immunoprecipitation-qPCR (MeRIP-qPCR)

A total of 6 × 10^7^ cells were lysed, frozen (liquid nitrogen), and melted. After centrifugation, the resulting supernatant was collected for the immunoprecipitation reaction. IGF2BP2 antibody or IgG was added and incubated overnight at 4 °C. After incubation with protein A/G immunomagnetic beads (Bimake, Houston, TX, USA) for 2 h, RNA immunoprecipitated by magnetic beads was extracted using TRIzol. For MeRIP-qPCR, RNA (extracted from 1 × 10^7^ cells) was resuspended in lysis buffer for subsequent immunoprecipitation reaction. Lysis buffer: 0.5% NP40, 150 mM KCl, 10 mM HEPES, 2 mM EDTA, 0.5 mM DTT, RNase inhibitors, and protease inhibitors.

### 4.10. Luciferase Reporter Assay

The 3′UTR of EGFR and PIK3R1 and the corresponding mutant were inserted into the psiCHECK™-2 vector (Promega, Madison, WI, USA), subsequently transfecting into HSC3-con/IGF2BP2-ko cells. Forty-eight hours after transfection, luciferase activity was measured using the Dual-Luciferase^®^ Reporter Assay System (Promega, WI, USA). Renilla luciferase activity was normalized to firefly luciferase activity.

### 4.11. Generation of Igf2bp2 Depletion Transgenic Mouse and Tongue SCC (TSCC) Induction

The *Igf2bp2* depletion transgenic mouse was generated through CRISPR/Cas9 technology by Cyagen (detailed in Figure S4). Primer sequences for PCR genotyping were listed in Table S4. Drinking water containing 4NQO (50 μg/mL, Sigma-Aldrich, St. Louis, MO, USA) was supplied to mice (8-week-old) for 18 weeks. When the weight of *Igf2bp2^wt^* mice dropped below 20 g, paired mice were euthanized. To assess the effect of CWI1-2, mice with lesions appearing on their tongues were intraperitoneally treated with saline or CWI1-2 (5.0 mg/kg) three times per week. The area of the most severe lesion on each mouse was measured weekly.

### 4.12. Single-Cell RNA Sequencing (scRNA-Seq) Data Source and Analysis

Raw counts of scRNA-seq from HNSCC tissues were acquired from the Gene Expression Omnibus database, accession number GSE234933 [58]. In our study, patients with fewer than 100 tumor cells, HPV-positive, or recurrence were excluded, and 22 patients remained (6 metastases and 16 primary HNSCC). Quality control and identification of major cell populations were performed with the same approach as the whole data. Cell subpopulations were re-identified based on known marker genes, i.e., MMP2 was used to identify CAFs. Seurat (version 5.1.0) was used to perform Uniform Manifold Approximation and Projection (UMAP) analysis and statistical computations.

### 4.13. Statistical Analysis

Statistical analysis was performed using GraphPad Prism 8 software (version 8.3.0). Student’s *t*-test was used for comparisons between two groups. Data are presented as mean ± SEM. *p* < 0.05 was considered statistically significant in all experiments.

## 5. Conclusions

In summary, our findings establish IGF2BP2 as the most prominently upregulated RBP in tumor cells and CAFs, highlighting its potential as a diagnostic and prognostic biomarker for OSCC. Furthermore, we elucidated a novel mechanism whereby IGF2BP2 accelerates OSCC progression through the stabilization of EGFR and PIK3R1 mRNA in an m6A-dependent manner, consequently activating the EGFR/PI3K/AKT signaling axis. Importantly, our results demonstrate that inhibition of IGF2BP2 not only effectively reverses the resistance of cetuximab-resistant cells to EGFR signaling blockade but also exhibits promising anti-OSCC efficacy in a CAF-infiltrated microenvironment. By elucidating the functional interplay among IGF2BP2, EGFR, and PIK3R1, our study deepens the understanding of the molecular mechanisms governing EGFR/PI3K/AKT activation in OSCC progression, potentially paving the way for novel anti-IGF2BP2 therapeutic strategies to overcome anti-EGFR resistance.

## Data Availability

The dataset supporting the conclusions of this article is available in the NCBI repository, https://dataview.ncbi.nlm.nih.gov/object/PRJNA1028396?reviewer=4mm3j1kjcdpkf9o7g8f5t18ukk (accessed on 29 July 2024).

## Supplementary Materials

The following supporting information can be downloaded at: https://www.mdpi.com/article/10.3390/ijms26093941/s1.

## Abbreviations

The following abbreviations are used in this manuscript:

CAFs: cancer-associated fibroblasts; CM-CAFs: conditioned-medium from CAFs; EGFR: epidermal growth factor receptor; FAP: fibroblast activation protein; GSEA: gene set enrichment analysis; HNSCC: head and neck squamous cell carcinoma; IF: immunofluorescence; IGF2BP2: insulin-like growth factor 2 mRNA binding protein 2; IHC: immunohistochemistry; LM: lymph node metastases; MeRIP-qPCR: methylated RNA immunoprecipitation-qPCR; NFs: normal fibroblasts; OSCC: oral squamous cell carcinoma; PIK3R1: phosphoinositide-3-kinase regulatory subunit 1; RBPs: RNA binding proteins; RIP-qPCR: RNA immunoprecipitation-qPCR; scRNA-seq: single-cell RNA sequencing; TSCC: tongue squamous cell carcinoma; UMAP: uniform manifold approximation and projection; α-SMA: α-smooth muscle actin.
