# Supplementary material for: Dual Disruption of EGFR/PI3K Signaling: IGF2BP2 Targeting Reverses Anti-EGFR Resistance in CAFs-Infiltrated Oral Squamous Cell Carcinoma
Source: Int J Mol Sci. 2025 Apr 22;26(9):3941. doi: 10.3390/ijms26093941 (PMC12072046; doi:10.3390/ijms26093941)

Supplementary Figures

Supplementary Figure S1:

Identification of the knockdown efficiency of IGF2BP2 in OSCC cells.

a. Expression of IGF2BP2 protein in HSC3 cells with IGF2BP2 knockdown. **b.** Expression of IGF2BP2 protein in Cal-27 cells with IGF2BP2 knockdown. **c.** Expression of IGF2BP2 protein in HSC3 cells with IGF2BP2 knockout.

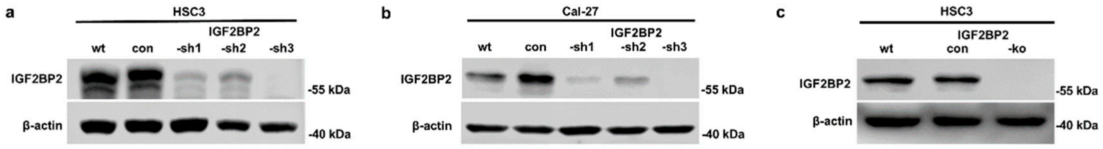

Supplementary Figure S2:

RNA and protein expression of α-SMA and FAP in NFs and CAFs.

a. RNA expression of α-SMA and FAP in NFs and CAFs. **b.** Protein expression of α-SMA in NFs and CAFs.

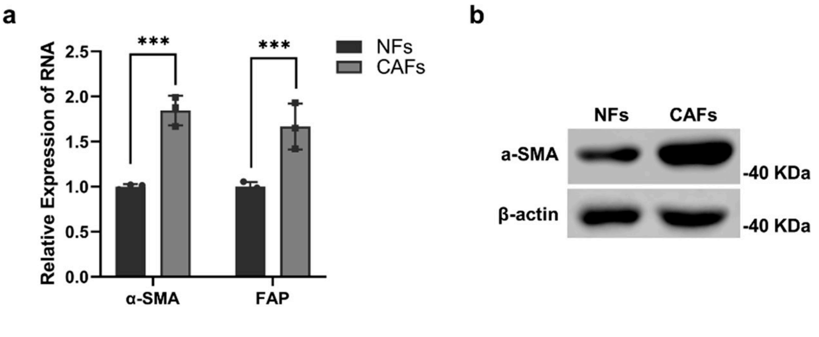

Supplementary Figure S3:

Generation of HSC3-IGF2BP2-ko cells.

a. Schematic depiction of the generation of HSC3-IGF2BP2-ko cells by CRPSPR-U™ targeting strategy. **b.** Genomic DNA from the HSC3-IGF2BP2-ko cells were characterized by DNA sequencing.

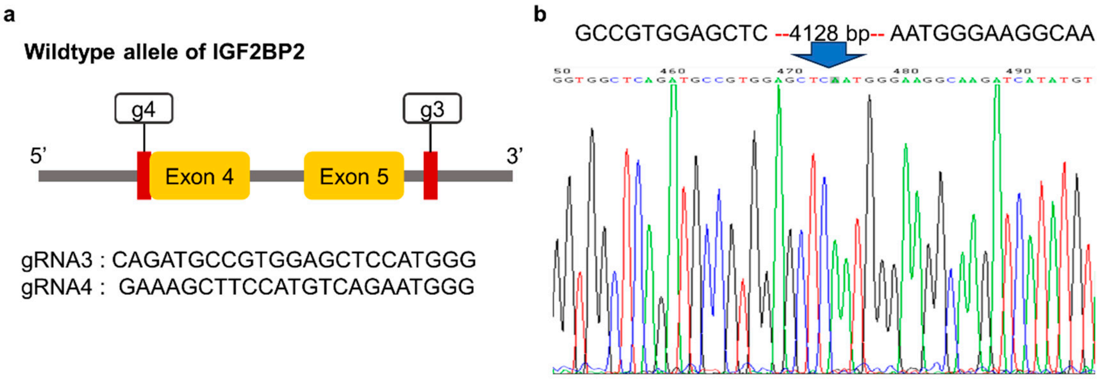

Supplementary Figure S4:

Generation of *Igf2bp2*$^{-/-}$ mice.

a. Schematic depiction of the generation of *Igf2bp2*$^{-/-}$ mice by CRISPR-Cas9 targeting strategy. **b.** Genomic DNA from the *Igf2bp2*$^{-/-}$ mice were characterized by DNA sequencing. **c-d.** PCR genotyping and western blotting results generated from *Igf2bp2*^{wt}, *Igf2bp2*$^{+/-}$, and *Igf2bp2*$^{-/-}$ mice.

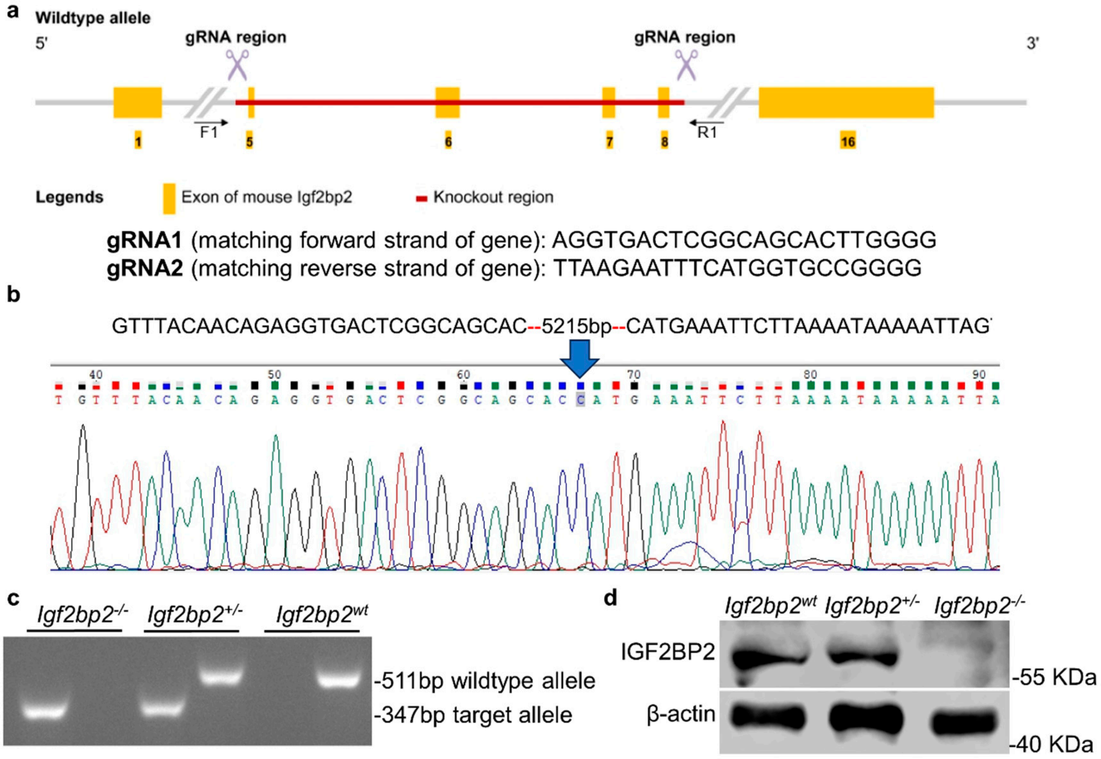

Supplement: Supplementary file 1 [file ijms-26-03941-s001.zip › Supplementary Figures.pdf]
