# Supplementary material for: Dual Disruption of EGFR/PI3K Signaling: IGF2BP2 Targeting Reverses Anti-EGFR Resistance in CAFs-Infiltrated Oral Squamous Cell Carcinoma
Source: Int J Mol Sci. 2025 Apr 22;26(9):3941. doi: 10.3390/ijms26093941 (PMC12072046; doi:10.3390/ijms26093941)
Supplement: Supplementary file 1 [file ijms-26-03941-s001.zip › Supplementary Tables.pdf]

Supplementary Tables

Supplementary Table S1:

Relationship between the different IGF2BP2 expression modes in OSCC and the patients' clinicopathological characteristics

Clinical characteristics	cases	IGF2BP2 expression		*P* value
		low	high	
**Gender**	196			0.9446
male		69	46	
female		49	32	
**Age(year)**	196			**0.0276***
≥60		50	21	
< 60		68	57	
**Tumor size(cm)**	97			0.4016
<4		26	10	
≥4		39	22	
**Nodal involvement**	196			**0.0296***
-		83	43	
+		35	35	
**M stage**	103			0.4599
0		67	30	
1		5	1	
**Clinical TNM**	101			0.9119
I-II		48	24	
III-IV		19	10	
**Grade stage**	195			**0.0017****
G1-2		111	61	
G3		7	16	

***P<0.05 **p<0.01**

Supplementary Table S2:

The sequences of IGF2BP2-sh1/2/3

	sequence
IGF2BP2 -sh1	A:5'-CCGGCGGATCTTTGGGAAACTGAAACTCGAGTTTCAGTTTCCCAAAGATCCGTTTTTG-3'
	B:5'-AATTCAAAAACGGATCTTTGGGAAACTGAAACTCGAGTTTCAGTTTCCCAAAGATCCG-3'
IGF2BP2 -sh2	A:5'-CCGGCAGTGCTGAGATAGAGATTATCTCGAGATAATCTCTATCTCAGCACTGTTTTTG-3'
	B:5'-AATTCAAAAACAGTGCTGAGATAGAGATTATCTCGAGATAATCTCTATCTCAGCACTG-3'
IGF2BP2 -sh3	A:5'-CCGGAGTGAAGCTGGAAGCGCATATCTCGAGATATGCGCTTCCAGCTTCACTTTTTTG-3'
	B:5'-AATTCAAAAAAGTGAAGCTGGAAGCGCATATCTCGAGATATGCGCTTCCAGCTTCACT-3'

Supplementary Table S3:

Primers sets for RT-qPCR

Gene	Primer Sequences
PIK3R1	F: 5'-ATATGTAGCAGAAAGGCACG-3'
	R: 5'-CAGCAAAACAAAACAGAAGC-3'
EGFR	F: 5'-CCTGGTATGGGTATGAAAGA-3'
	R: 5'-TGGTCATCCTCCTGTGAG-3'
KRAS	F: 5'-CAGGCTCAGGACTTAGCAAGA-3'
	R: 5'-AGGCATCATCAACACCCAGAT-3'
HSP90AA1	F: 5'-GCCCAGAGTGCTGAATACCC-3'
	R: 5'-TAACAGGTGCCCTGCTTCTC-3'
β-actin	F: 5'-ACCAACTGGGACGACATGGAGAAA-3'
	R: 5'-TAGCACAGCCTGGATAGCAACGTA-3'
α-SMA	F: 5'-AAAGCAAGTCCTCCAGCGTT-3'
	R: 5'-GCTTCACAGGATTCCCGTCT-3'
FAP	F: 5'-TCTGGAAAAATGAAGACTTGGGT-3'
	R: 5'-ACTCTTGAAGGGCGTAAGACA-3'

Supplementary Table S4:

Primers for PCR genotyping

Gene	Primer Sequences	PCR products length
***Igf2bp2*-wt-allele**	F: 5'-GACTGATTGGCAAAGAAGGCAG-3'<br>R: 5'-ACAGCTAACGCTGCCCTTAAC-3'	**511 bp**
***Igf2bp2*-mutated-allele**	F: 5'-CTTCCACCCATTTTTCCTCTTTAG-3'<br>R: 5'-ACAGCTAACGCTGCCCTTAAC-3'	**347 bp**
